# Acupuncture does not work and has no place in science-based medicine

**DOI:** 10.1073/pnas.2613544123

**Published:** 2026-05-07

**Authors:** Piet Borst, Anton Berns, Roel Nusse

**Affiliations:** ^a^The Netherlands Cancer Institute, Amsterdam 1066 CX, The Netherlands; ^b^Howard Hughes Medical Institute, Stanford University School of Medicine, Stanford, CA 94305

In the PNAS of March 4, 2026, Lynne Peeples presented a news feature on the “science behind acupuncture” (1). We find this article unbalanced. It lacks a critical analysis of what acupuncture is and ignores whether acupuncture is more than an effective placebo.

Historically, acupuncture arose in China at a time when there was no detailed knowledge of the anatomy and physiology of the human body. The basic concepts of acupuncture, the acupuncture points and meridians, are philosophical constructs and have no relation to modern knowledge of human bodies, whatever proponents may claim (2). As an example, Peeples quotes an acupuncturist who is “regulating the flow of qi and blood, balancing yin and yang, and harmonizing organ function through the meridians.” This is not science but a text that does not belong in science-based medicine.

But even if the theory has no basis in state-of-the-art knowledge of pathophysiology, could acupuncture work? Is it more than a placebo? Placing needles into patients generates a significant placebo effect, especially when the acupuncturist is a believer in the positive effects. It is evidently tough to control for the dramatic effects of the mere process of needling. Nevertheless, some carefully designed trials with adequate controls and sham acupuncture treatments have been published (2–6) and these have given unambiguous results: The placebo effect is indeed highly significant, while the difference between real and sham acupuncture is negligible (3–5).

Unfortunately, promoting alternative medical treatments is not without risk, as ineffective alternative treatments may displace effective verified ones. Acupuncture needles in patients have side-effects as well. Poor disinfection of needles may spread infections and needles may damage vital body parts (6). For instance, needling can cause lungs to collapse (pneumothorax) and this is not rare (6, 7). Promoting alternative medical treatments by an established scientific journal such as the PNAS is not without negative side-effects: Clinicians are constantly confronted with empty claims by practitioners of alternative treatments and clinicians’ tasks are further complicated when articles in the PNAS can be quoted to bolster such claims.
